# Ethyl 2-(5-cyclo­hexyl-3-methyl­sulfinyl-1-benzofuran-2-yl)acetate

**DOI:** 10.1107/S1600536811024925

**Published:** 2011-07-02

**Authors:** Pil Ja Seo, Hong Dae Choi, Byeng Wha Son, Uk Lee

**Affiliations:** a Department of Chemistry, Dongeui University, San 24 Kaya-dong Busanjin-gu, Busan 614-714, Republic of Korea; b Department of Chemistry, Pukyong National University, 599-1 Daeyeon 3-dong, Nam-gu, Busan 608-737, Republic of Korea

## Abstract

In the title compound, C_19_H_24_O_4_S, the cyclo­hexyl ring adopts a chair conformation. In the crystal, mol­ecules are linked by weak inter­molecular C—H⋯O hydrogen bonds. The O atom of the sulfinyl group is disordered over two orientations with site-occupancy factors of 0.875 (4) and 0.125 (4).

## Related literature

For the pharmacological activity of benzofuran compounds, see: Aslam *et al.* (2009 ▶); Galal *et al.* (2009 ▶); Khan *et al.* (2005 ▶). For natural products with benzofuran rings, see: Akgul & Anil (2003 ▶); Soekamto *et al.* (2003 ▶). For structural studies of related ethyl 2-(3-methyl­sulfinyl-1-benzofuran-2-yl) acetate derivatives, see: Choi *et al.* (2007**a* ▶,b* ▶; 2009 ▶).
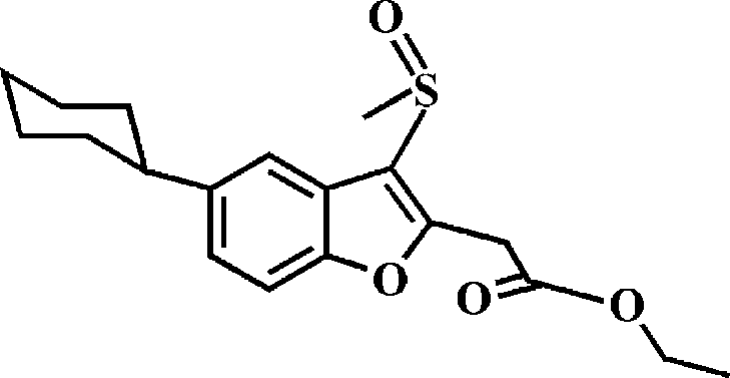


## Experimental

### 

#### Crystal data

C_19_H_24_O_4_S
*M* *_r_* = 348.45
Monoclinic, 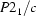

*a* = 16.1065 (7) Å
*b* = 4.8485 (2) Å
*c* = 22.3653 (11) Å
β = 91.010 (2)°
*V* = 1746.29 (14) Å^3^
*Z* = 4
Mo *K*α radiation
μ = 0.21 mm^−1^
*T* = 173 K
0.28 × 0.20 × 0.18 mm

#### Data collection

Bruker SMART APEXII CCD diffractometer
Absorption correction: multi-scan (*SADABS*; Bruker, 2009 ▶) *T*_min_ = 0.945, *T*_max_ = 0.964
15388 measured reflections
3804 independent reflections
2917 reflections with *I* > 2σ(*I*)
*R*_int_ = 0.049

#### Refinement

*R*[*F* ^2^ > 2σ(*F* ^2^)] = 0.052
*wR*(*F* ^2^) = 0.132
*S* = 1.02
3804 reflections
229 parameters
67 restraints
H-atom parameters constrained
Δρ_max_ = 0.56 e Å^−3^
Δρ_min_ = −0.38 e Å^−3^

### 

Data collection: *APEX2* (Bruker, 2009 ▶); cell refinement: *SAINT* (Bruker, 2009 ▶); data reduction: *SAINT*; program(s) used to solve structure: *SHELXS97* (Sheldrick, 2008 ▶); program(s) used to refine structure: *SHELXL97* (Sheldrick, 2008 ▶); molecular graphics: *ORTEP-3* (Farrugia, 1997 ▶) and *DIAMOND* (Brandenburg, 1998 ▶); software used to prepare material for publication: *SHELXL97*.

## Supplementary Material

Crystal structure: contains datablock(s) global, I. DOI: 10.1107/S1600536811024925/bv2187sup1.cif

Structure factors: contains datablock(s) I. DOI: 10.1107/S1600536811024925/bv2187Isup2.hkl

Supplementary material file. DOI: 10.1107/S1600536811024925/bv2187Isup3.cml

Additional supplementary materials:  crystallographic information; 3D view; checkCIF report

## Figures and Tables

**Table 1:** Hydrogen-bond geometry (Å, °)

*D*—H⋯*A*	*D*—H	H⋯*A*	*D*⋯*A*	*D*—H⋯*A*
C14—H14*B*⋯O4*A*^i^	0.99	2.60	3.479 (3)	149
C15—H15*B*⋯O2^ii^	0.99	2.56	3.437 (3)	147

Symmetry codes: (i) 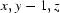; (ii) 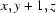.

**Fig. 1.**
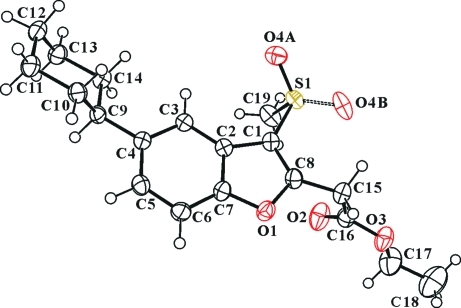
The molecular structure of the title compound with the atom numbering scheme. Displacement ellipsoids are drawn at the 50% probability level. H atoms are presented as a small spheres of arbitrary radius. The O atom of sulfinyl group is disordered over two positions with site-occupancy factors, from refinement of 0.875 (3) (part A) and 0.125 (4) (part B).

**Fig. 2.**
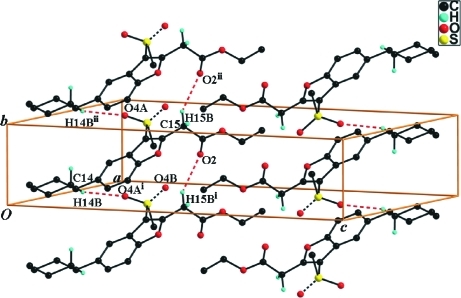
A view of the C—H···O interactions (dotted lines) in the crystal structure of the title compound. [Symmetry codes: (i) x, y - 1, z; (ii) x, y + 1, z.]

## supplementary crystallographic information

### Comment

Recently, many compounds containing a benzofuran moiety have drawn much attention owing to their valuable pharmacological properties such as antibacterial and antifungal, antitumor and antiviral, and antimicrobial activities (Aslam *et al.* , 2009, Galal *et al.* , 2009, Khan *et al.* , 2005). These compounds occur in a wide range of natural products (Akgul & Anil, 2003; Soekamto *et al.* , 2003). As a part of our ongoing study of the substituent effect on the solid state structures of ethyl 2-(3-methylsulfinyl-1-benzofuran-2-yl)acetate analogues (Choi *et al.*, 2007**a*,b*; 2009), we report herein on the crystal structure of the title compound

In the title molecule (Fig. 1), the benzofuran unit is essentially planar, with a mean deviation of 0.011 (2) Å from the least-squares plane defined by the nine constituent atoms. The cyclohexyl ring is in the chair form. The O atom of  sulfinyl group is disordered over two positions with site-occupancy factors, from refinement of 0.875 (3) (part A) and 0.125 (4) (part B). The molecular packing (Fig. 2) is stabilized by weak intermolecular C—H···O hydrogen bonds; the first one between a cyclohexyl H atom and the O atom of the sulfinyl group (Table 1; C14—H14B···O4A^i^), and the second one between an H atom of the benzylic methylene group and the O atom of the carbonyl group (Table 1; C15—H15B···O2^ii^).

### Experimental

77% 3-chloroperoxybenzoic acid (224 mg, 1.0 mmol) was added in small portions to a stirred solution of ethyl 2-(5-cyclohexyl-3-methylsulfanyl-1-benzofuran-2-yl)acetate (298 mg, 0.9 mmol) in dichloromethane (40 mL) at 273 K. After being stirred at room temperature for 4h, the mixture was washed with saturated sodium bicarbonate solution and the organic layer was separated, dried over magnesium sulfate, filtered and concentrated at reduced pressure. The residue was purified by column chromatography (hexane–ethyl acetate, 1:2 v/v) to afford the title compound as a colorless solid [yield 70%, m.p. 360–361 K; *R*_f_ = 0.41 (hexane–ethyl acetate, 1:2 v/v)]. Single crystals suitable for X-ray diffraction were prepared by slow evaporation of a solution of the title compound in ethyl acetate at room temperature.

### Refinement

All H atoms were positioned geometrically and refined using a riding model, with C—H = 0.95 Å for aryl, 1.00 Å for methine, 0.99 Å for methylene and 0.98 Å for methyl H atoms, respectively.  *U*_iso_(H)  = 1.2*U*_eq_(C)  for aryl,  methine,  methylene,  and 1.5*U*_eq_(C) for methyl H atoms. The O atom of sulfinyl group is disordered over two  positions with site-ccupancy factors, from refinement of 0.875 (4) (part A)  and 0.125 (4) (part B). The S—O distances were restrained to 0.001 Å using command SADI and DELU.

### Crystal data

**Table:**

C_19_H_24_O_4_S	*F*(000) = 744
*M**_r_* = 348.45	*D*_x_ = 1.325 Mg m^−^^3^
Monoclinic, *P*2_1_/*c*	Mo *K*α radiation, λ = 0.71073 Å
Hall symbol: -P 2ybc	Cell parameters from 3275 reflections
*a* = 16.1065 (7) Å	θ = 2.2–26.5°
*b* = 4.8485 (2) Å	µ = 0.21 mm^−^^1^
*c* = 22.3653 (11) Å	*T* = 173 K
β = 91.010 (2)°	Block, colourless
*V* = 1746.29 (14) Å^3^	0.28 × 0.20 × 0.18 mm
*Z* = 4	

### Data collection

**Table:**

Bruker SMART APEXII CCD diffractometer	3804 independent reflections
Radiation source: rotating anode	2917 reflections with *I* > 2σ(*I*)
graphite multilayer	*R*_int_ = 0.049
Detector resolution: 10.0 pixels mm^-1^	θ_max_ = 27.0°, θ_min_ = 1.3°
φ and ω scans	*h* = −20→19
Absorption correction: multi-scan (*SADABS*; Bruker, 2009)	*k* = −6→6
*T*_min_ = 0.945, *T*_max_ = 0.964	*l* = −28→28
15388 measured reflections	

### Refinement

**Table:**

Refinement on *F*^2^	Primary atom site location: structure-invariant direct methods
Least-squares matrix: full	Secondary atom site location: difference Fourier map
*R*[*F*^2^ > 2σ(*F*^2^)] = 0.052	Hydrogen site location: difference Fourier map
*wR*(*F*^2^) = 0.132	H-atom parameters constrained
*S* = 1.02	*w* = 1/[σ^2^(*F*_o_^2^) + (0.0537*P*)^2^ + 1.3587*P*] where *P* = (*F*_o_^2^ + 2*F*_c_^2^)/3
3804 reflections	(Δ/σ)_max_ < 0.001
229 parameters	Δρ_max_ = 0.56 e Å^−^^3^
67 restraints	Δρ_min_ = −0.38 e Å^−^^3^

### Special details

**Table:**

Geometry. All esds (except the esd in the dihedral angle between two l.s. planes) are estimated using the full covariance matrix. The cell esds are taken into account individually in the estimation of esds in distances, angles and torsion angles; correlations between esds in cell parameters are only used when they are defined by crystal symmetry. An approximate (isotropic) treatment of cell esds is used for estimating esds involving l.s. planes.
Refinement. Refinement of F^2^ against ALL reflections. The weighted R-factor wR and goodness of fit S are based on F^2^, conventional R-factors R are based on F, with F set to zero for negative F^2^. The threshold expression of F^2^ > 2sigma(F^2^) is used only for calculating R-factors(gt) etc. and is not relevant to the choice of reflections for refinement. R-factors based on F^2^ are statistically about twice as large as those based on F, and R- factors based on ALL data will be even larger.

### Fractional atomic coordinates and isotropic or equivalent isotropic displacement parameters (Å^2^)

**Table:**

	*x*	*y*	*z*	*U*_iso_*/*U*_eq_	Occ. (<1)
S1	0.36614 (3)	0.95799 (12)	0.29116 (3)	0.03096 (17)	
O1	0.18470 (9)	0.6531 (3)	0.38601 (6)	0.0325 (4)	
O2	0.38323 (12)	0.5969 (4)	0.44371 (8)	0.0532 (5)	
O3	0.36351 (11)	0.9044 (4)	0.51602 (7)	0.0443 (5)	
O4A	0.35467 (11)	1.0448 (4)	0.22886 (8)	0.0392 (6)	0.875 (4)
O4B	0.3933 (9)	1.160 (2)	0.3361 (5)	0.057 (4)	0.125 (4)
C1	0.27598 (12)	0.7963 (4)	0.31809 (9)	0.0266 (5)	
C2	0.22021 (12)	0.6069 (4)	0.28840 (9)	0.0253 (4)	
C3	0.21169 (12)	0.5003 (4)	0.23082 (9)	0.0262 (5)	
H3	0.2479	0.5575	0.2001	0.031*	
C4	0.14931 (12)	0.3085 (4)	0.21906 (9)	0.0257 (5)	
C5	0.09692 (13)	0.2284 (5)	0.26516 (10)	0.0316 (5)	
H5	0.0548	0.0962	0.2567	0.038*	
C6	0.10392 (13)	0.3335 (5)	0.32258 (10)	0.0327 (5)	
H6	0.0679	0.2773	0.3535	0.039*	
C7	0.16600 (13)	0.5242 (5)	0.33243 (9)	0.0283 (5)	
C8	0.25217 (13)	0.8178 (5)	0.37491 (10)	0.0297 (5)	
C9	0.13521 (13)	0.1884 (4)	0.15724 (9)	0.0269 (5)	
H9	0.1225	−0.0119	0.1624	0.032*	
C10	0.05931 (13)	0.3205 (5)	0.12640 (9)	0.0308 (5)	
H10A	0.0100	0.2924	0.1515	0.037*	
H10B	0.0686	0.5216	0.1227	0.037*	
C11	0.04212 (14)	0.1998 (6)	0.06457 (10)	0.0379 (6)	
H11A	0.0255	0.0042	0.0686	0.045*	
H11B	−0.0046	0.3007	0.0453	0.045*	
C12	0.11783 (14)	0.2186 (5)	0.02536 (10)	0.0362 (5)	
H12A	0.1304	0.4148	0.0173	0.043*	
H12B	0.1058	0.1270	−0.0134	0.043*	
C13	0.19283 (15)	0.0828 (5)	0.05528 (10)	0.0358 (5)	
H13A	0.2420	0.1071	0.0299	0.043*	
H13B	0.1825	−0.1175	0.0595	0.043*	
C14	0.21039 (13)	0.2080 (5)	0.11680 (9)	0.0317 (5)	
H14A	0.2261	0.4041	0.1121	0.038*	
H14B	0.2579	0.1106	0.1359	0.038*	
C15	0.28665 (14)	0.9685 (5)	0.42765 (10)	0.0338 (5)	
H15A	0.2408	1.0187	0.4544	0.041*	
H15B	0.3131	1.1413	0.4141	0.041*	
C16	0.34984 (14)	0.7987 (5)	0.46210 (10)	0.0347 (5)	
C17	0.42421 (19)	0.7598 (7)	0.55380 (13)	0.0591 (8)	
H17A	0.4054	0.5687	0.5612	0.071*	
H17B	0.4784	0.7519	0.5337	0.071*	
C18	0.4327 (2)	0.9083 (9)	0.61010 (14)	0.0775 (11)	
H18A	0.4544	1.0937	0.6026	0.116*	
H18B	0.4711	0.8088	0.6369	0.116*	
H18C	0.3783	0.9225	0.6288	0.116*	
C19	0.42883 (14)	0.6559 (5)	0.28894 (12)	0.0403 (6)	
H19A	0.4039	0.5228	0.2610	0.060*	
H19B	0.4325	0.5739	0.3290	0.060*	
H19C	0.4846	0.7046	0.2757	0.060*	

### Atomic displacement parameters (Å^2^)

**Table:**

	*U*^11^	*U*^22^	*U*^33^	*U*^12^	*U*^13^	*U*^23^
S1	0.0292 (3)	0.0237 (3)	0.0402 (3)	−0.0018 (2)	0.0030 (2)	0.0002 (3)
O1	0.0286 (8)	0.0414 (9)	0.0276 (8)	−0.0026 (7)	0.0032 (6)	−0.0066 (7)
O2	0.0576 (12)	0.0484 (11)	0.0531 (11)	0.0198 (10)	−0.0138 (9)	−0.0149 (10)
O3	0.0490 (10)	0.0465 (11)	0.0368 (9)	0.0081 (9)	−0.0149 (8)	−0.0089 (8)
O4A	0.0363 (10)	0.0412 (12)	0.0402 (11)	−0.0020 (9)	0.0010 (8)	0.0072 (9)
O4B	0.058 (9)	0.030 (7)	0.083 (9)	−0.013 (6)	−0.001 (8)	−0.008 (7)
C1	0.0227 (10)	0.0267 (11)	0.0302 (10)	0.0026 (9)	−0.0007 (8)	−0.0023 (9)
C2	0.0211 (9)	0.0254 (11)	0.0294 (10)	0.0025 (9)	0.0000 (8)	−0.0003 (9)
C3	0.0242 (10)	0.0272 (11)	0.0271 (10)	0.0014 (9)	−0.0001 (8)	−0.0002 (9)
C4	0.0251 (10)	0.0253 (11)	0.0266 (10)	0.0027 (9)	−0.0009 (8)	−0.0011 (9)
C5	0.0279 (11)	0.0319 (12)	0.0350 (11)	−0.0035 (10)	−0.0028 (9)	−0.0014 (10)
C6	0.0267 (10)	0.0405 (13)	0.0312 (11)	−0.0049 (10)	0.0050 (9)	−0.0006 (11)
C7	0.0251 (10)	0.0318 (12)	0.0279 (10)	0.0028 (9)	−0.0016 (8)	−0.0043 (10)
C8	0.0245 (10)	0.0308 (12)	0.0336 (11)	−0.0002 (9)	0.0002 (9)	−0.0048 (10)
C9	0.0313 (11)	0.0222 (11)	0.0270 (10)	−0.0006 (9)	−0.0023 (8)	−0.0014 (9)
C10	0.0248 (10)	0.0342 (12)	0.0335 (11)	−0.0021 (10)	−0.0017 (9)	−0.0003 (10)
C11	0.0323 (12)	0.0469 (15)	0.0341 (12)	−0.0067 (11)	−0.0080 (9)	0.0010 (11)
C12	0.0384 (12)	0.0431 (14)	0.0270 (11)	−0.0051 (11)	−0.0041 (9)	−0.0024 (11)
C13	0.0416 (13)	0.0358 (13)	0.0301 (11)	0.0010 (11)	0.0030 (10)	−0.0049 (11)
C14	0.0297 (11)	0.0350 (12)	0.0305 (11)	0.0053 (10)	−0.0016 (9)	−0.0043 (10)
C15	0.0338 (11)	0.0365 (13)	0.0309 (11)	0.0016 (10)	−0.0017 (9)	−0.0073 (10)
C16	0.0322 (12)	0.0371 (13)	0.0346 (12)	−0.0027 (11)	−0.0018 (9)	−0.0044 (11)
C17	0.0637 (18)	0.0570 (18)	0.0557 (17)	0.0124 (16)	−0.0278 (15)	−0.0027 (15)
C18	0.077 (2)	0.102 (3)	0.0525 (18)	0.025 (2)	−0.0274 (16)	−0.0068 (19)
C19	0.0274 (11)	0.0317 (13)	0.0620 (16)	0.0040 (10)	0.0077 (11)	0.0001 (12)

### Geometric parameters (Å, °)

**Table:**

S1—O4B	1.463 (2)	C10—H10A	0.9900
S1—O4A	1.4641 (19)	C10—H10B	0.9900
S1—C1	1.765 (2)	C11—C12	1.517 (3)
S1—C19	1.780 (2)	C11—H11A	0.9900
O1—C8	1.375 (3)	C11—H11B	0.9900
O1—C7	1.380 (2)	C12—C13	1.520 (3)
O2—C16	1.193 (3)	C12—H12A	0.9900
O3—C16	1.325 (3)	C12—H12B	0.9900
O3—C17	1.460 (3)	C13—C14	1.526 (3)
C1—C8	1.338 (3)	C13—H13A	0.9900
C1—C2	1.439 (3)	C13—H13B	0.9900
C2—C7	1.387 (3)	C14—H14A	0.9900
C2—C3	1.392 (3)	C14—H14B	0.9900
C3—C4	1.391 (3)	C15—C16	1.510 (3)
C3—H3	0.9500	C15—H15A	0.9900
C4—C5	1.399 (3)	C15—H15B	0.9900
C4—C9	1.514 (3)	C17—C18	1.455 (4)
C5—C6	1.385 (3)	C17—H17A	0.9900
C5—H5	0.9500	C17—H17B	0.9900
C6—C7	1.377 (3)	C18—H18A	0.9800
C6—H6	0.9500	C18—H18B	0.9800
C8—C15	1.487 (3)	C18—H18C	0.9800
C9—C14	1.527 (3)	C19—H19A	0.9800
C9—C10	1.533 (3)	C19—H19B	0.9800
C9—H9	1.0000	C19—H19C	0.9800
C10—C11	1.523 (3)		
			
O4B—S1—O4A	119.5 (6)	C10—C11—H11B	109.3
O4B—S1—C1	107.5 (6)	H11A—C11—H11B	108.0
O4A—S1—C1	111.19 (10)	C11—C12—C13	111.10 (19)
O4B—S1—C19	114.0 (6)	C11—C12—H12A	109.4
O4A—S1—C19	105.79 (12)	C13—C12—H12A	109.4
C1—S1—C19	96.58 (11)	C11—C12—H12B	109.4
C8—O1—C7	105.52 (16)	C13—C12—H12B	109.4
C16—O3—C17	116.1 (2)	H12A—C12—H12B	108.0
C8—C1—C2	107.52 (19)	C12—C13—C14	110.96 (19)
C8—C1—S1	122.73 (17)	C12—C13—H13A	109.4
C2—C1—S1	129.54 (16)	C14—C13—H13A	109.4
C7—C2—C3	119.77 (19)	C12—C13—H13B	109.4
C7—C2—C1	104.63 (18)	C14—C13—H13B	109.4
C3—C2—C1	135.6 (2)	H13A—C13—H13B	108.0
C4—C3—C2	118.8 (2)	C13—C14—C9	111.91 (18)
C4—C3—H3	120.6	C13—C14—H14A	109.2
C2—C3—H3	120.6	C9—C14—H14A	109.2
C3—C4—C5	119.3 (2)	C13—C14—H14B	109.2
C3—C4—C9	121.74 (19)	C9—C14—H14B	109.2
C5—C4—C9	118.92 (19)	H14A—C14—H14B	107.9
C6—C5—C4	122.8 (2)	C8—C15—C16	112.1 (2)
C6—C5—H5	118.6	C8—C15—H15A	109.2
C4—C5—H5	118.6	C16—C15—H15A	109.2
C7—C6—C5	116.3 (2)	C8—C15—H15B	109.2
C7—C6—H6	121.9	C16—C15—H15B	109.2
C5—C6—H6	121.9	H15A—C15—H15B	107.9
C6—C7—O1	126.1 (2)	O2—C16—O3	124.2 (2)
C6—C7—C2	123.0 (2)	O2—C16—C15	125.1 (2)
O1—C7—C2	110.79 (18)	O3—C16—C15	110.7 (2)
C1—C8—O1	111.53 (18)	C18—C17—O3	108.3 (2)
C1—C8—C15	133.0 (2)	C18—C17—H17A	110.0
O1—C8—C15	115.31 (19)	O3—C17—H17A	110.0
C4—C9—C14	114.16 (17)	C18—C17—H17B	110.0
C4—C9—C10	110.85 (17)	O3—C17—H17B	110.0
C14—C9—C10	110.00 (18)	H17A—C17—H17B	108.4
C4—C9—H9	107.2	C17—C18—H18A	109.5
C14—C9—H9	107.2	C17—C18—H18B	109.5
C10—C9—H9	107.2	H18A—C18—H18B	109.5
C11—C10—C9	112.27 (19)	C17—C18—H18C	109.5
C11—C10—H10A	109.2	H18A—C18—H18C	109.5
C9—C10—H10A	109.2	H18B—C18—H18C	109.5
C11—C10—H10B	109.2	S1—C19—H19A	109.5
C9—C10—H10B	109.2	S1—C19—H19B	109.5
H10A—C10—H10B	107.9	H19A—C19—H19B	109.5
C12—C11—C10	111.51 (18)	S1—C19—H19C	109.5
C12—C11—H11A	109.3	H19A—C19—H19C	109.5
C10—C11—H11A	109.3	H19B—C19—H19C	109.5
C12—C11—H11B	109.3		
			
O4B—S1—C1—C8	−13.8 (7)	C2—C1—C8—O1	0.8 (2)
O4A—S1—C1—C8	−146.31 (19)	S1—C1—C8—O1	−174.34 (14)
C19—S1—C1—C8	103.9 (2)	C2—C1—C8—C15	176.7 (2)
O4B—S1—C1—C2	172.3 (7)	S1—C1—C8—C15	1.6 (4)
O4A—S1—C1—C2	39.7 (2)	C7—O1—C8—C1	−0.3 (2)
C19—S1—C1—C2	−70.1 (2)	C7—O1—C8—C15	−176.97 (19)
C8—C1—C2—C7	−0.9 (2)	C3—C4—C9—C14	21.3 (3)
S1—C1—C2—C7	173.75 (17)	C5—C4—C9—C14	−160.1 (2)
C8—C1—C2—C3	179.9 (2)	C3—C4—C9—C10	−103.6 (2)
S1—C1—C2—C3	−5.4 (4)	C5—C4—C9—C10	75.0 (2)
C7—C2—C3—C4	−1.0 (3)	C4—C9—C10—C11	−178.72 (18)
C1—C2—C3—C4	178.1 (2)	C14—C9—C10—C11	54.1 (2)
C2—C3—C4—C5	0.1 (3)	C9—C10—C11—C12	−54.8 (3)
C2—C3—C4—C9	178.68 (19)	C10—C11—C12—C13	55.2 (3)
C3—C4—C5—C6	0.5 (3)	C11—C12—C13—C14	−55.9 (3)
C9—C4—C5—C6	−178.2 (2)	C12—C13—C14—C9	56.5 (3)
C4—C5—C6—C7	0.0 (3)	C4—C9—C14—C13	179.72 (18)
C5—C6—C7—O1	−179.3 (2)	C10—C9—C14—C13	−54.9 (2)
C5—C6—C7—C2	−1.0 (3)	C1—C8—C15—C16	−88.6 (3)
C8—O1—C7—C6	178.2 (2)	O1—C8—C15—C16	87.2 (2)
C8—O1—C7—C2	−0.3 (2)	C17—O3—C16—O2	0.5 (4)
C3—C2—C7—C6	1.5 (3)	C17—O3—C16—C15	−179.5 (2)
C1—C2—C7—C6	−177.8 (2)	C8—C15—C16—O2	16.1 (3)
C3—C2—C7—O1	−179.89 (18)	C8—C15—C16—O3	−163.9 (2)
C1—C2—C7—O1	0.8 (2)	C16—O3—C17—C18	178.5 (3)

### Hydrogen-bond geometry (Å, °)

**Table:**

*D*—H···*A*	*D*—H	H···*A*	*D*···*A*	*D*—H···*A*
C14—H14B···O4A^i^	0.99	2.60	3.479 (3)	149
C15—H15B···O2^ii^	0.99	2.56	3.437 (3)	147

Symmetry codes: (i) *x*, *y*−1, *z*; (ii) *x*, *y*+1, *z*.
